# Evaluation of the Incidence and Risk Factors of Deep Vein Thrombosis in Lower Limb Surgery in the North Indian Population

**DOI:** 10.7759/cureus.93701

**Published:** 2025-10-02

**Authors:** Amrit Goyal, Ravikant Rohela, Hari Singh, Mayur Gupta, Vikash Singh, Rajat Kapoor

**Affiliations:** 1 Department of Orthopaedic Surgery, Sarojini Naidu Medical College, Agra, IND; 2 Department of Radiodiagnosis, Sarojini Naidu Medical College, Agra, IND; 3 Department of Orthopaedics, Sarojini Naidu Medical College, Agra, IND

**Keywords:** anticoagulant, color doppler ultrasonography, deep vein thrombosis (dvt), hypertension, pulmonary embolism, type 2 diabetes

## Abstract

Background: In patients undergoing lower limb surgery, deep vein thrombosis (DVT) is a serious postoperative complication. If not detected and treated right away, it can cause serious morbidity and even death. The purpose of this study is to determine the prevalence of DVT and the risk factors linked to it in patients in the North Indian population undergoing lower limb surgery.

Methods: Patients undergoing various lower limb surgical procedures at a tertiary care hospital in North India were the subjects of this prospective observational study. Duplex Doppler ultrasonography was used to assess patients and keep an eye out for DVT symptoms after surgery within 3 to 5 days.

Results: The present study was conducted on 65 individuals, all of whom underwent orthopedic surgical procedures. The overall incidence of DVT in the study population was 24.6%, with the remaining 75.4% of subjects showing no evidence of DVT. Factors such as advanced age, prolonged immobilization, previous history of DVT, and presence of comorbidities like “diabetes and hypertension” were found to be strongly associated with an increased risk of developing DVT.

Conclusion: The study highlights the importance of early identification and prevention of DVT in patients undergoing lower limb surgery. The overall DVT incidence of 24.6%, particularly in patients with intertrochanteric and femoral neck fractures, reinforces the need for thromboprophylaxis in this population. Risk stratification and use of prophylactic measures such as anticoagulants and early mobilization can play a crucial role in reducing the incidence of DVT in the North Indian population.

## Introduction

Deep vein thrombosis (DVT) stands as a medical condition characterized by the formation of clots inside the deep veins, most commonly affecting the legs. DVT not only impedes postoperative recovery but also poses a significant risk of life-threatening complications, such as pulmonary embolism (PE), wherein a dislodged thrombus migrates to the pulmonary vasculature, causing obstruction and potentially fatal cardiopulmonary compromise. A comprehensive understanding of the incidence, risk factors, and preventive strategies for DVT is imperative to mitigate its impact, particularly in high-risk cohorts such as patients undergoing lower limb surgical interventions [1].

In India, DVT affects one person out of every 1000 members of the general population. The incidence of DVT after major lower limb surgery in the occurrence rate of DVT in Indian surgical patients matches Western statistics. Among reported cases, 43% are idiopathic, 52% occur following a precipitating event, and 6% of patients experience recurrence. PE, a life-threatening complication of the annual occurrence of DVT, affects between 39 and 115 people per 100,000 members of the population. The condition stands as the leading reason for unexpected patient death among hospitalized patients. The mortality rate of untreated PE is as high as 30%, but with early diagnosis and appropriate treatment, PE-related deaths can be reduced to less than 1% [2,3]. DVT is a manifestation of venous thromboembolism (VTE), encompassing the formation of thrombi within the deep venous system, most frequently in the femoral, popliteal, and iliac veins. The pathogenesis of DVT is underpinned by Virchow’s triad, which includes venous stasis, endothelial injury, and hypercoagulability [4,5]. Systemic factors, including malignancy, pregnancy, and the use of exogenous hormones (e.g., oral contraceptives or hormone replacement therapy), contribute to a hypercoagulable state, amplifying the risk of DVT [6-8]. Lower limb surgeries, including total knee arthroplasty, total hip arthroplasty, internal fixation of fractures, and soft tissue reconstructive procedures, are associated with a significantly elevated risk of DVT. These procedures often involve extensive soft tissue dissection, prolonged operative times, and the use of tourniquets, all of which contribute to venous stasis and endothelial trauma. Additionally, the release of tissue factor and other procoagulant substances during surgical trauma further exacerbates the hypercoagulable state [9]. In the North Indian population, the risk is compounded by a high prevalence of metabolic syndromes, such as diabetes and obesity, as well as cultural practices that may delay postoperative ambulation [10,11]. In the context of lower limb surgery, PE is a leading cause of perioperative mortality, underscoring the critical importance of early diagnosis and prophylactic measures to prevent DVT [12,13]. This article aims to evaluate the incidence and delineate the risk factors of DVT in patients undergoing lower limb surgery within the North Indian population. By conducting a detailed analysis of demographic variables, surgical practices, and postoperative outcomes, this study seeks to elucidate the epidemiology of DVT in this region.

## Materials and methods

This prospective study was conducted at the Department of Orthopedics, Sarojini Naidu Medical College, Agra. Patients were selected from those attending the emergency and outpatient department (OPD) of Orthopedics. The study included all patients undergoing lower limb surgeries that required postoperative immobilization. The study was conducted over a period from May 2023 to October 2024. The sample size was calculated based on the incidence of DVT in lower limb surgeries using the formula:

n=Z2⋅p⋅q/L2

Where: p=7.2% (expected incidence of DVT in orthopedic surgery, as referenced from [14]); q=100−p

 Z=1.645 (for a 90% significance level)

 L=5% (margin of error)

Power of study = 80%

The minimum sample size required was calculated to be 65 patients. This was a prospective follow-up study.

Postoperative assessment

All patients underwent a handheld Doppler study of the deep venous system of both lower limbs between postoperative days 3 and 5. Confirmation of DVT was done using duplex scanning. Detailed clinical examinations were conducted to assess the presence of DVT. The relevant data were collected using the following methods: Detailed History: Comprehensive patient histories were taken, including any prior medical conditions, medications, and risk factors for DVT.

Both Wells criteria for DVT and PE are clinical prediction rules that assess pre-test probability of VTE. While the DVT score emphasizes local limb signs (swelling, tenderness, edema), the PE score incorporates systemic features (tachycardia, hemoptysis, immobilization) and the likelihood of an alternative diagnosis. Together, they streamline diagnostic work-up, reduce unnecessary imaging, and support evidence-based management of thromboembolic disorders (Table 1).

**Table 1:** Comparison of Wells Criteria for DVT and PE The Wells score is based on several clinical features and risk factors. Each feature is assigned a specific point value. DVT: Deep vein thrombosis; PE: pulmonary embolism

Criteria	DVT Wells Score	PE Wells Score
Active cancer (treatment ongoing/within 6 months or palliative)	1	1
Paralysis, paresis, or recent plaster immobilization of lower extremities	1	–
Bedridden >3 days or major surgery within 12 weeks (with anesthesia)	1	Immobilization ≥3 days or surgery within 4 weeks → +1.5
Localized tenderness along deep vein system	1	–
Entire leg swollen	1	–
Calf swelling >3 cm compared with asymptomatic leg	1	–
Pitting edema (confined to symptomatic leg)	1	–
Collateral superficial veins (non-varicose)	1	–
Previous DVT	1	Previous DVT or PE → +1.5
Heart rate >100/min	–	1.5
Hemoptysis	–	1
Clinical signs/symptoms of DVT	–	3
Alternative diagnosis as likely/more likely than DVT/PE	–2	–3
Scoring Interpretation	≥3 = High probability; 1–2 = Moderate; ≤0 = Low	>6 = High probability; 2–6 = Moderate; <2 = Low (Simplified: >4 = PE likely; ≤4 = PE unlikely)

Surgical data

Information on the nature of the surgery, duration, blood loss, and blood transfusions was recorded from the patient's operation notes.

Hematological investigations

Complete hemogram, liver function tests, renal function tests, lipid profile (HDL, LDL, total cholesterol), and coagulation profile (PT, aPTT, INR) were conducted.

Imaging studies

Handheld Doppler and venous duplex scanning were used to confirm the presence of DVT.

Inclusion criteria

All patients who underwent elective or emergency lower limb operations and were admitted to S.N. Medical College, Agra, were included in the study.

Exclusion criteria

Patients who underwent cardiac or vascular operations, Patients who had taken anticoagulants such as warfarin, aspirin, or clopidogrel during the week before hospital admission, Patients with uncorrectable coagulopathy, Patients on heparin therapy, and Patients with a previous history of DVT were excluded. Statistical analysis was performed using the latest available version of SPSS and MS Excel. Multivariate analysis was employed for the statistical study of the data. Continuous variables conforming to a normal distribution were expressed as mean ± standard deviation. Counting data were expressed as numbers and percentages. A p-value of less than 0.05 was considered statistically significant.

Assessment of PE in patients with suspected or confirmed DVT begins with clinical evaluation for symptoms such as dyspnea, chest pain, hemoptysis, or syncope. Risk stratification tools like the Wells Criteria are used to determine pre-test probability. Laboratory tests, particularly D-dimer, are useful in low to intermediate-risk patients, while high-risk cases proceed directly to imaging. CT pulmonary angiography is the gold standard, with V/Q scan as an alternative. Echocardiography, ECG, and leg vein ultrasound provide supportive evidence, especially in unstable patients. Timely diagnosis and anticoagulation are crucial for reducing morbidity and mortality.

## Results

The present study was conducted on 65 individuals, all of whom underwent orthopedic surgical procedures. The mean age of the participants was 53.14 years, with a standard deviation of 16.11 years, indicating a moderately wide age distribution within the sample. Age-wise categorization revealed that the 51-60 years age group comprised the largest proportion (24.6%) of the study population, followed by individuals aged 61-70 years (16.9%).

In terms of gender distribution, there was a slightly higher proportion of female participants (52.3%) compared to male participants (47.7%), resulting in a near equal gender representation. The analysis of comorbid conditions among the subjects showed that hypertension (HTN) was the most commonly reported medical condition, affecting 41.5% of the individuals. Type 2 diabetes mellitus (T2DM) was the second most prevalent comorbidity, present in 36.9% of the sample. Postoperative Doppler ultrasonography was conducted to assess the presence of deep vein thrombosis. The results showed that a majority of the participants (84.6%) had a negative Doppler study, while 15.4% tested positive for DVT. These findings served as a diagnostic basis for evaluating the incidence of DVT in the study population.

The overall incidence of DVT in the study population was 24.6%, with the remaining 75.4% of subjects showing no evidence of DVT. A stratified analysis provided deeper insights into the association between DVT occurrence and various demographic and clinical variables. A striking 87.5% of subjects with HTN developed DVT, compared to only 12.5% among non-hypertensive subjects. This difference was found to be statistically significant, underscoring HTN as a major risk factor for DVT in this population. Similarly, among subjects with T2DM, 75.0% were diagnosed with DVT, while only 25.0% of non-diabetics developed the condition. The strength and significance of this association were also confirmed in multivariate regression analysis (Table 2).

**Table 2:** Assessment of risk factors for deep venous thrombosis using multivariate logistic regression analysis Assessment of risk factors for deep venous thrombosis using multivariate logistic regression analysis identified several potential risk factors associated with deep vein thrombosis (DVT). Among the variables assessed, female gender, hypertension, and type 2 diabetes mellitus were found to be statistically significant predictors. Female gender was associated with nearly three times higher odds of developing DVT (OR: 2.79, p = 0.025). Hypertension also showed a strong and significant association (OR: 3.28, p < 0.001), indicating it as an important risk factor. Notably, type 2 diabetes mellitus emerged as the most significant predictor, with individuals having over nine times increased odds of DVT (OR: 9.25, p < 0.001). Although individuals aged over 50 years had higher odds (OR: 1.474), the association did not reach statistical significance (p = 0.176) (Z-values rounded to two decimal places). The asterisk (*) indicates that the result is statistically significant at the chosen threshold (commonly p < 0.05).

Variable	OR	95% CI	p-value	Z-value
Age (>50 years)	1.474	0.841–2.584	0.176	1.35
Female gender	2.79	0.55–5.52	0.025*	2.24
Hypertension	3.28	1.26–2.95	< 0.001*	3.49
Type 2 diabetes mellitus	9.25	2.51–3.17	< 0.001*	6.88

The incidence of DVT based on the type of treatment they received is as above table: 25% (20 subjects) underwent bipolar prosthesis, 25% (eight subjects) received femur intramedullary (I/L) nail fixation, 24% (25 subjects) were treated with a proximal femoral nail (PFN), 30% (10 subjects) had a total hip replacement, and not reported (two subjects) who underwent total knee replacement (TKR). This distribution highlights the varying frequency of these different orthopedic treatments among the study participants (Table 3).

**Table 3:** Treatment-wise distribution of study subjects and incidence of DVT Treatment-wise distribution of study subjects shows the most common treatments were PFN (proximal femoral nail) (38.5%) and bipolar hemiarthroplasty (30.8%). Other treatments included Femur I/L nail (12.3%), THR (total hip replacement) (15.4%), and TKR (total knee replacement) (3.1%). DVT: Deep vein thrombosis

	Frequency	Incidence of DVT
Bipolar	20 (30.8%)	5 (25%)
Femur I/L Nail	8 (12.3%)	2 (25%)
PFN	25 (38.5%)	6 (24%)
THR	10 (15.5%)	3 (30%)
TKR	2 (3.1%)	0

## Discussion

DVT remains a significant postoperative complication in lower limb surgeries, contributing to increased morbidity and healthcare burden. While global data on DVT incidence and risk factors are well documented, regional variations in genetic predisposition, lifestyle factors, and surgical practices may influence outcomes in specific populations. This study evaluates the incidence and risk factors of DVT following lower limb surgeries in the North Indian population, where limited comprehensive data exists. Our findings aim to bridge this knowledge gap and identify population-specific risk patterns that could optimize thromboprophylaxis strategies in this demography. The frequency and percentage of participants were categorized into various age groups within a study sample of 65 individuals. The highest representation is seen in the 51-60 age group with 16 participants, making up 24.6% of the total. Additionally, those aged over 70 years represent 18.5% of the sample, with 12 individuals. Overall, the distribution indicates that the majority of the participants are middle-aged to elderly, with the mean age being 53.14 years and a standard deviation of 16.11 years, suggesting a wide age range among the study subjects. Similar results were reported by Bagaria et al. (2006) [15], where the average age of patients undergoing major orthopedic surgery was 64.2 years.

The distribution of study subjects based on whether they received a blood transfusion. Out of the total participants, 44 individuals, accounting for 67.7% of the sample, did not receive a blood transfusion during the study period. In contrast, 21 participants, representing 32.3% of the total, did undergo blood transfusion. This data suggests that the majority of the study subjects did not require a transfusion, while approximately one-third of them did, indicating a notable proportion of the population experiencing clinical conditions necessitating blood transfusion. Mioc et al. (2018) [16] reported that a significant proportion of patients (69.15%) undergoing lower limb fracture surgeries required blood transfusions either intraoperatively or postoperatively. The relationship between HTN and DVT among the study subjects. Out of the total subjects without HTN, 36 individuals (73.5%) did not have DVT, while two individuals (12.5%) had DVT. Conversely, among the subjects with HTN, 13 individuals (26.5%) did not have DVT, whereas 14 individuals (87.5%) had DVT. This data indicates a significant association between HTN and the presence of DVT, with a higher prevalence of DVT observed in individuals with HTN. Similar findings were reported by Zhang et al. [17], who observed that HTN was significantly associated with an increased risk of developing DVT.

The association between HTN and DVT among subjects with and without T2DM. Among subjects without T2DM, 37 individuals (75.5%) did not have HTN, while 12 individuals (24.5%) did have HTN. In contrast, among those with T2DM, four individuals (25.0%) did not have HTN, while a significant 12 individuals (75.0%) had HTN. This data suggests a higher prevalence of HTN among individuals with T2DM compared to those without, indicating a potential relationship between T2DM and HTN in the context of DVT. A study by Piazza et al. (2012) [18] found that 79.4% of patients with diabetes also have HTN, compared to 48.2% of patients without diabetes.

Analysis of several significant risk factors for DVT through multivariate logistic regression analysis

Among the variables examined, female gender, HTN, and T2DM emerged as strong and statistically significant predictors of DVT. The association between female gender and increased risk of DVT (OR: 2.79, p = 0.025) is notable, though somewhat unexpected. While previous studies have often reported a higher incidence of DVT in males, certain female-specific factors such as hormonal therapy, oral contraceptive use, pregnancy, and the postpartum period may contribute to this elevated risk. Further investigation is warranted to determine whether these factors were present or disproportionately represented in the female subgroup of this study. HTN also demonstrated a significant association with DVT (OR: 3.28, p < 0.001). Chronic HTN can lead to vascular endothelial damage and reduced blood flow, potentially promoting thrombus formation. This finding aligns with existing literature, which has identified HTN as a contributor to VTE, particularly in the presence of additional cardiovascular risk factors. The most striking finding was the strong association between T2DM and DVT (OR: 9.25, p < 0.001). T2DM is known to induce a prothrombotic state through mechanisms such as endothelial dysfunction, increased platelet aggregation, and elevated levels of coagulation factors. The high odds ratio observed suggests that T2DM may be a critical and independent driver of DVT risk, underscoring the importance of aggressive metabolic control and thromboprophylaxis in diabetic populations.

The study included 65 patients undergoing lower limb orthopedic surgery; 16 patients (24.6%) developed DVT. No cases of pulmonary thromboembolism or mortality were reported. Despite the relatively high incidence of DVT, early detection and monitoring likely prevented serious complications, highlighting the importance of thromboprophylaxis in high-risk populations.

## Conclusions

In conclusion, the incidence of DVT in our North Indian cohort was 24.6%, with a significant proportion missed by single Doppler screening. Risk factors strongly associated with DVT included older age, female sex, emergency surgery, perioperative blood transfusion, and especially HTN. The high prevalence of comorbidities like HTN and diabetes, coupled with the predominance of high-risk fracture types, underscores the need for vigilant perioperative risk assessment.

Our findings advocate for a more proactive approach toward DVT prevention in lower limb surgeries, particularly among elderly, diabetic and hypertensive patients. Universal pharmacologic thromboprophylaxis may not be feasible or safe in all patients; hence, individual risk stratification based on demographic and clinical parameters becomes crucial. Future studies with larger sample sizes and longitudinal follow-up are needed to validate these observations and refine DVT prevention protocols in Indian surgical settings.
